# Advances in Microbial Biotechnology for Sustainable Alternatives to Petroleum-Based Plastics: A Comprehensive Review of Polyhydroxyalkanoate Production

**DOI:** 10.3390/microorganisms12081668

**Published:** 2024-08-13

**Authors:** Silvia González-Rojo, Ana Isabel Paniagua-García, Rebeca Díez-Antolínez

**Affiliations:** 1 Department of Chemistry and Applied Physics, Chemical Engineering Area, Campus de Vegazana s/n, University of León, 24071 León, Spain; 2 Centro de Biocombustibles y Bioproductos, Instituto Tecnológico Agrario de Castilla y León (ITACyL), Polígono Agroindustrial del Órbigo p. 2-6, Villarejo de Órbigo, 24358 León, Spain; pangaran@itacyl.es (A.I.P.-G.); dieantre@itacyl.es (R.D.-A.)

**Keywords:** PHA, microbial biotechnology, genetic engineering, bioplastics, polyhydroxyalkanoates

## Abstract

The industrial production of polyhydroxyalkanoates (PHAs) faces several limitations that hinder their competitiveness against traditional plastics, mainly due to high production costs and complex recovery processes. Innovations in microbial biotechnology offer promising solutions to overcome these challenges. The modification of the biosynthetic pathways is one of the main tactics; allowing for direct carbon flux toward PHA formation, increasing polymer accumulation and improving polymer properties. Additionally, techniques have been implemented to expand the range of renewable substrates used in PHA production. These feedstocks are inexpensive and plentiful but require costly and energy-intensive pretreatment. By removing the need for pretreatment and enabling the direct use of these raw materials, microbial biotechnology aims to reduce production costs. Furthermore, improving downstream processes to facilitate the separation of biomass from culture broth and the recovery of PHAs is critical. Genetic modifications that alter cell morphology and allow PHA secretion directly into the culture medium simplify the extraction and purification process, significantly reducing operating costs. These advances in microbial biotechnology not only enhance the efficient and sustainable production of PHAs, but also position these biopolymers as a viable and competitive alternative to petroleum-based plastics, contributing to a circular economy and reducing the dependence on fossil resources.

## 1. Introduction

The production of petroleum-based plastics has grown exponentially in recent decades. Global plastics production increased from 1.5 million metric tons (Mt) in the 1950s [1] to 370.5 Mt in 2018, reaching 400.3 Mt in 2022, with a positive compound annual growth rate through 2032 [2]. This massive increase has led to serious environmental and health consequences from the uncontrolled release of plastic waste. Plastic pollution is now ubiquitous in the environment, with plastic particles found in even the most remote regions of the planet. Estimating the amount of plastic waste in the world’s oceans is challenging, but various estimates agree that more than 8 million tons of plastic waste enters the oceans each year [3]. This plastic pollution harms marine ecosystems and wildlife, and it can take centuries to degrade. Moreover, micro- and nanoplastics produced by the weathering, oxidative damage and mechanical stress of plastic products can have devastating effects on animals and ultimately humans [4]. In fact, micro- and nanoplastics have been found in drinking water, food and even human blood, raising concerns about potential health impacts. In addition, they could be transmitted through the food chain, ultimately affecting humans who consume these contaminated animals. These compounds have been linked to adverse long-term human health effects due to their ability to bioaccumulate in tissues and cause oxidative stress, which can affect organism survival, growth, reproduction and energy metabolism [5].

In response to the growing plastic pollution crisis, governments around the world are implementing policies to reduce plastic waste and encourage the development of more sustainable alternatives. The European Union has banned certain single-use plastic products and set targets for recycling and reducing plastic waste. Many countries have also implemented bans or taxes on plastic bags and other single-use plastic items. In particular, the European Union has implemented the Circular Economy Action Plan as part of its European Green Deal, which aims to make Europe the first carbon-neutral continent by 2050. The EU’s Circular Economy Action Plan is a comprehensive set of legislative and non-legislative measures aimed at transforming the European economy from a linear to a circular model, promoting systemic change through innovation and investment. To meet these new regulatory requirements and consumer demand for more green products, there is an urgent need to develop alternatives to traditional petroleum-based plastics. Bioplastics, which are derived from renewable biomass sources (“bio-based”), biodegradable and produced by biological processes, or a combination of these, offer a promising solution [6]. However, users should be cautious when using this terminology, as in many cases the term “bioplastics” is often used for products that do not meet the above characteristics. For example, bioplastic is sometimes used to refer to some polymers that are made from renewable sources but are not biodegradable. On the other hand, the term is used to refer to plastics derived from fossil sources that are biodegradable. Bioplastics, as bio-based and biodegradable polymers, offer a promising alternative to reduce the environmental impact of plastic waste. Examples of bioplastics include polylactic acid (PLA) made from corn starch, cellulose-based plastics made from wood pulp or agricultural waste, and polyhydroxyalkanoates (PHAs) produced by bacteria [7].

The rapid growth of plastics production and the resulting environmental and health consequences have highlighted the need for new sustainable alternatives to traditional plastics. Recent advances in microbial biotechnology have significantly improved the production of PHAs. Microorganisms, such as *Cupriavidus necator* and *Halomonas* species, have been genetically engineered to enhance their ability to accumulate PHAs [8]. These microorganisms can convert a variety of renewable carbon sources, including agricultural wastes and industrial by-products, into PHAs, making the process more sustainable and cost-effective [9]. In addition, innovative bioprocessing techniques, such as fed-batch and continuous fermentation, have been developed to optimize microbial PHA production, increase yields and reduce production costs [10].

Furthermore, the use of synthetic biology has opened new avenues for the production of tailor-made PHAs with specific properties. By modifying the metabolic pathways of PHA-producing bacteria, researchers can now produce PHAs with different monomer compositions, resulting in bioplastics with varied mechanical and thermal properties suitable for diverse applications [11]. For instance, PHAs with enhanced flexibility, tensile strength or thermal stability can be produced to meet specific industry needs, such as packaging, agriculture or biomedical applications [12].

This review focuses specifically on PHAs and evaluates how innovative microbial biotechnologies have improved the production of these biopolymers. A comprehensive bibliographic review of scientific articles and book chapters, mostly published in the last 10 years, was conducted using primary databases such as Scopus, Pubmed and Google Scholar. This review aims to provide a perspective on the progress made and on how microbial biotechnology can improve the production, economics and competitiveness of these biopolymers, positioning them as key players in the transition away from petroleum-based plastics.

## 2. PHAs: Types, Synthesis and Industrial Production

PHAs are a family of biodegradable bioplastics produced intracellularly by microorganisms as an energy reserve in the form of granules when environmental conditions are adverse, mainly when there is an excess of carbon source and a nutrient limitation related to nitrogen or phosphorus sources in the media [8,13,14]. These biopolymers are biodegradable, biocompatible and non-toxic, making them attractive alternatives to traditional plastics.

### 2.1. Types of PHAs

**Figure 1:**
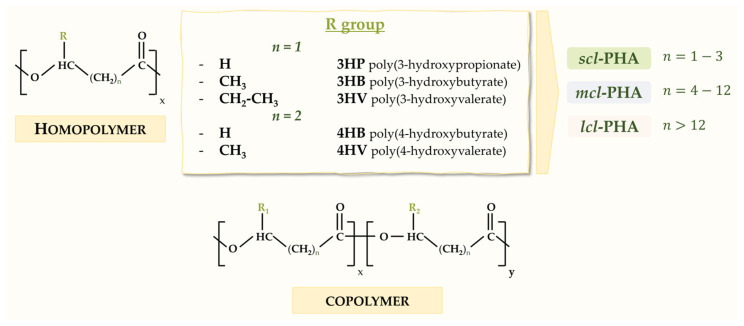
Basic chemical structure of PHA monomers in homopolymers and copolymers with specific examples of PHA monomers.

PHAs are made of ester-bonded (R)-hydroxyalkanoate monomers (Figure 1). According to the number of carbon atoms in their monomer units, PHAs are generally classified into three types: *short-chain length* PHAs (*scl*-PHAs) consisting of 3–5 carbon atoms per monomer unit, *medium-chain length* PHAs (*mcl*-PHAs) that have 6–14 carbon atoms per monomer unit and *long-chain length* PHAs (*lcl*-PHAs) with more than 14 carbon atoms per monomer unit [15] (Figure 1). PHAs exhibit distinct properties depending on their composition and structure, and possess unique mechanical and thermal properties such as high tensile strength, flexibility and resistance to heat and chemicals [16]. The ability to form copolymers, namely polymers with monomer units of different carbon chain lengths, means that the versatility of these bioplastics will be wide due to the variability in their basic properties. The most common commercial PHAs are the homopolymer poly(3-hydroxybutyrate) (PHB) and the copolymer poly(3-hydroxybutyrate-*co*-3-hydroxyvalerate) (PHBV), both of which are considered as *scl*-PHAs [17]. The side chain size of PHAs influences some of the inherent properties of the polymer and significantly contributes to its molecular structures. In this way, as the number of carbon atoms in the side chain increases, the resulting polymer is more elastic, with a lower crystallinity and melting point. For example, *scl*-PHAs are brittle with a high crystallinity and melting temperature, whereas *mcl*-PHAs are considered elastomers with a low crystallinity and melting temperature [18].

### 2.2. Biosynthesis of PHAs

**Figure 2:**
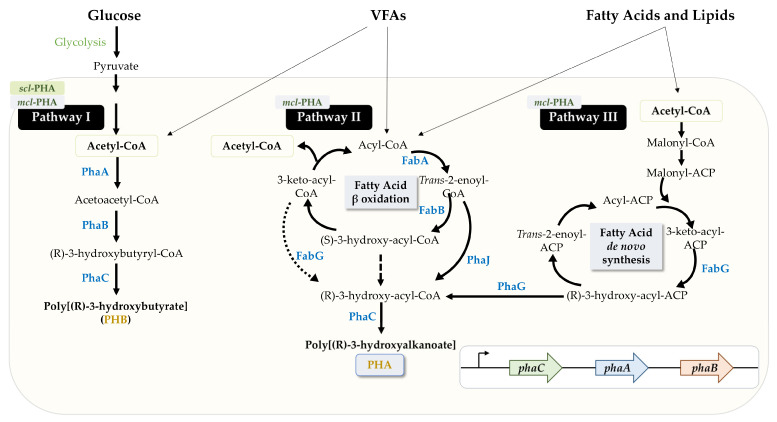
Main biosynthetic pathways for PHA production. Some enzymes are indicated in specific steps: PhaA: 3-ketothyolase; PhaB: NADPH-dependent acetoacetyl-CoA reductase; PhaC: PHA synthase; PhaG: (R)-3-hydroxyacyl-ACP-CoA transferase; PhaJ: enoyl CoA hydratase; FabA: 3-ketoacyl-CoA thiolase; FabB: enoyl-CoA hydratase; FabG: 3-ketoacyl-CoA reductase; VFAs: volatile fatty acids; *scl*-PHA: short chain-length PHA; *mcl*-PHA: medium chain-length PHA. The *phaCAB* operon in *Cupriavidus necator* is also shown.

There are several distinct species of microorganisms that produce PHAs, including several members of Arquea and approximately 250 different species of bacteria, both Gram-positive and Gram-negative [19,20]. Nevertheless, the most commonly used species in the commercial production of PHAs are those belonging to the genera *Cupriavidus*, *Pseudomonas* and *Bacillus*, with *C. necator* being one of the most commonly used species [13,21]. *Pseudomonas putida,* with its ability to produce *mcl*-PHAs, is another well-known example [22], or *Halomonas* spp., an extremophile microorganism that can produce PHAs under high salinity conditions [23]. Depending on the type of PHA-producing microorganisms and the carbon source, three different metabolic pathways involved in PHA production can be distinguished (Figure 2). Pathway I is the classical pathway for PHB synthesis, one of the most studied PHAs, and involves three key enzymes: PhaA, PhaB and PhaC. Acetoacetyl-CoA is formed by the condensation of two molecules of acetyl-CoA catalyzed by PhaA (3-ketothyolase). PhaB (NADPH-dependent acetoacetyl-CoA reductase) converts this substrate to (R)-3-hydroxybutyryl-CoA, and the assembly of this monomer into a PHB chain is orchestrated by the enzyme PhaC (PHA synthase) [17] (Figure 2). Pathway II is associated with fatty acid β-oxidation, in which an acyl-CoA-type precursor is converted to (R)-3-hydroxyacyl-CoA trough the catalytic action of the enzyme PhaJ (enoyl-CoA hydratase). PhaC can then act on this substrate to produce PHAs [24]. In Pathway III, which is related to the *de novo* fatty acid synthesis pathway, acetyl-CoA is converted to (R)-3-hydroxyacyl-ACP, which is then used as a substrate by the enzyme PhaG ((R)-3-hydroxyacyl-ACP-CoA transferase) to form (R)-3-hydroxyacyl-CoA, which is then incorporated into the expanding PHA chain [25].

Since the monomers incorporated into the growing PHA chain are crucial to the physical and thermal properties of the resulting biopolymer, PHA synthase plays a key role in these characteristics by determining the monomers that are added to the polymer chain [26]. PHA synthase can be distinguished by substrate specificity or subunit composition. Based on this, four classes could be categorized: Class I, III and IV for *scl*-PHA synthesis and Class II for *mcl*-PHA production [27,28], using precursors from lipid *de novo* synthesis or long-chain fatty acid catabolism. In addition, Class I and II are single-unit enzymes, whereas Class III and IV contain two subunits [26].

### 2.3. Industrial Production of PHAs

The production process of PHAs presents a number of common steps that occur in each mode of operation. On the one hand, the production starts with the microbial culture, which usually has an initial phase in which an increase in cell biomass is sought, and a final phase in which the accumulation of the polymer occurs, usually due to the deprivation of some essential nutrients such as nitrogen or phosphorus, while there is an excess of the carbon source. On the other hand, the production of the biopolymer may end with the downstream phase, where the goal is to recover the polymer with the highest purity. Since it is a biocompound that is not excreted to the outside, it is necessary to separate the microbial biomass from the culture broth and dry it, then recover the bioplastic from the inside of the cell using different methods, some of which are able to solubilize the polymer and others that precipitate it. Traditional methods include the use of halogenated solvents, which offer very high extraction yields and purity, but are currently frowned upon not only due to the health problems they cause, but also due to the negative environmental impact they generate [29]. Other more sustainable methods based on the use of green solvents are being researched and developed [30].

The industrial production and commercialization of PHAs is still struggling due to high production costs that make the biopolymer about 3–4 times more expensive than petroleum-based plastics [9,21]. The main reasons for this high cost include the following: (i) the use of high-purity substrates, which can account for up to 45% of the total production cost [8,9,27]; (ii) batch or fed-batch production, which is less efficient and more costly than continuous mode operation [31]; (iii) the large volumes of solvents used and the labor required for downstream processing, which substantially increase the cost of production [32].

However, recent research has concentrated on identifying alternatives to reduce PHA production costs. In this sense, one of the most studied and booming proposals, also favored by the new green policies, is the valorization of wastes and by-products. The use of renewable substrates opens up the possibility of obtaining PHA bioplastics from a wide range of raw materials that vary greatly in their composition. In this sense, a variety of renewable wastes have been used, mostly from the agroindustrial sector, consisting of high carbon-rich wastes such as molasses from the sugar industry [33], fruit wastes [34], dairy processing wastes [35], waste from oil or biodiesel plants [36,37], lignocellulosic materials such as sugarcane bagasse [38] and urban and industrial wastewater [39,40]. These feedstocks are characterized as inexpensive and abundant carbon sources that can significantly reduce the cost of production of PHAs. In most cases, the type of waste used determines the type of microorganism that performs the biotransformation given the complexity of its composition. For this reason, the selection of the producing microorganism and the selected culture conditions are crucial for the efficient production of PHAs, so the selection must be made based on the ability to accumulate a large amount of biopolymer, if possible from different carbon sources, and the culture conditions must also be optimized to maximize production [24]. Another important aspect is the ability to grow microorganisms in open systems, without sterile conditions, to make the process even cheaper, as the cost of sterilization can be up to 30% of the total production cost at the commercial scale [41]. In this regard, it is worth mentioning the extremophilic microorganisms, especially those capable of growing in hypersaline environments, which have been in the spotlight in recent years as promising for the production of PHA biopolymers from renewable substrates under non-sterile situations. In addition, they are easily manipulated by microbial biotechnology to promote PHA efficiency, which has led them to be considered as platform organisms for next-generation industrial biotechnology (NIGB) [8]. In addition to reducing production costs due to their culture without sterile conditions, they could contribute to reducing the costs associated with polymer recovery, since less expensive methods such as hypoosmotic shock with salt-deficient water [29] could facilitate the extraction of PHAs.

## 3. Overcoming the Limitations of Industrial PHA Production with Microbial Biotechnology

Even with new developments that attempt to address the main limitations of the industrial production of PHAs, further efforts are needed to make the commercialization of these bioplastics competitive against traditional plastics. Microbial biotechnology offers important solutions to overcome the challenges of industrial production. For example, genetic engineering has allowed for the modification of microorganisms to improve their ability to produce PHAs from renewable carbon sources, thereby increasing the range of substrates that can be biotransformed by a PHA-producing microorganism. In some cases, this has allowed for the elimination of waste pretreatment processes, improving the sustainability of the process. In addition, by modifying the metabolic flux of the microorganisms, it has been possible to direct the production toward the desired type of bioplastics, improving the amount accumulated and the properties of the polymer. In particular, the ability to design biopolymers with a wider range of monomers, including those with aromatic side chains, represents a significant opportunity to expand the applications of PHAs and make them even more competitive in the marketplace. Other strategies have made it possible to modify the cell size to increase the amount of biopolymer accumulated, or even to excrete the bioplastic outside the cell to simplify the recovery process, thereby increasing productivity and reducing production costs. These advances show how microbial biotechnology can be the key to opening the door to new developments that will make bioplastic production more efficient, sustainable and economically feasible.

### 3.1. Brief Introduction to Microbial Biotechnology Techniques for PHA Production

Bacterial conjugation and electroporation are among the most classical technologies used to genetically modify microorganisms to improve PHA production. Bacterial conjugation involves the transfer of genetic material between bacteria through direct cell-to-cell contact. This method has been used to introduce exogenous DNA into PHA-producing bacteria, allowing for the incorporation of beneficial genes that enhance PHA production [8]. However, this method only allows for the introduction of plasmid DNA. Electroporation, on the other hand, uses electrical pulses to create temporary pores in the cell membranes of bacteria, allowing foreign DNA to enter the cells and providing a broader and faster genome-wide genetic modification. This technique has been widely used to modify PHA-producing microorganisms by introducing plasmids containing genes to improve bioplastic synthesis [42]. While effective, these methods often have limitations such as low transformation efficiencies and random integration of exogenous DNA, which can lead to unpredictable genetic outcomes [43,44]. Furthermore, these techniques can be cumbersome and time-consuming, making them less suitable for large-scale industrial applications.

Other molecular genetic methods widely used in prokaryotes include bacteriophage transduction and transposon mutagenesis. The first technique uses bacterial viruses to transfer DNA from one bacterial cell to another, facilitating the insertion of genes of interest, while the second uses mobile genetic elements that can insert themselves randomly into the genome, causing mutations [8]. Other systems, such as the double crossover gene deletion system, allow for the removal of specific sequences of bacterial DNA by homologous recombination [8].

One of the most widely used emerging technologies in NGIB is the Clustered Regularly Interspaced Short Palindromic Repeats/CRISPR-associated protein (CRISPR/Cas) system, which has emerged as a powerful and precise genome editing tool that is revolutionizing the field of microbial biotechnology. This technology allows for targeted modifications of specific genomic sequences, enabling precise genetic alterations with high efficiency and accuracy.

**Figure 3:**
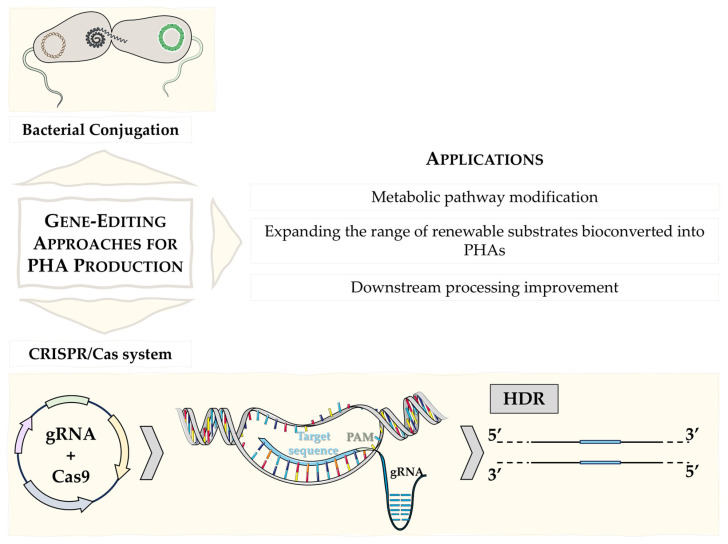
Application of some of the most commonly used genetic engineering techniques to improve PHA production. The graph shows a representative picture of two genetic engineering systems using bacterial conjugation and the CRISPR/Cas9 system via homologous recombination (HDR) pathway.

The CRISPR/Cas9 technology consists of two main components: the Cas9 protein, a DNA endonuclease derived from *Streptococcus pyogenes* that acts as a molecular scissors, and a guide RNA (gRNA), 20 nucleotides long and complementary to the target DNA sequence, located 5′ of the NGG (protospacer adjacent motif sequence—PAM sequence), which directs the Cas9 protein to the specific DNA sequence to be edited [8,45,46]. Double-strand breaks (DSBs) are made in the DNA by the Cas9 protein when it is directed to the target sequence by the gRNA. DSBs in DNA can be naturally repaired in the cell by various mechanisms, allowing for the insertion, deletion or modification of genetic material at the target site. After Cas9 cut, the error-prone NHEJ (Non-Homologous DNA End Joining) pathway can be activated, which favors indel-type mutations, or the HDR (Homology Directed Repair) pathway can be used to introduce a modified sequence by swapping sequences between two similar DNA molecules [47] (Figure 3).

However, before these two components can take action, the first step is to induce the expression of an exogenous recombinase, followed by co-transformation with the recombinant DNA that will serve as a guide and the plasmid containing the CRISPR system (Cas9 and gRNA) [48]. Within the target DNA sequence, each protospacer is always associated with a PAM sequence, which depends on the CRISPR system used. The implementation of all these elements can vary depending on the strategy, with all these elements potentially found in a single plasmid or not. Finally, CRISPRi (CRISPR interference) is a technique that inhibits gene expression without altering the DNA sequence by using a variant of CRISPR/Cas9 that binds to specific DNA sequences to block transcription [8].

One of the major advantages of the CRISPR/Cas system over traditional methods is its ability to achieve specific site modifications with high precision, minimizing off-target activity. This precision is particularly important in PHA-producing bacteria, where specific genetic changes can have a significant impact on metabolic pathways and improve PHA production. For example, researchers have used CRISPR/Cas to eliminate genes that compete for substrate or energy resources, thereby redirecting more resources to PHA synthesis [49]. In this context, CRISPR/Cas technology enables the rational design of microbial cell factories with optimized metabolic pathways, leading to higher yields and lower energy production costs, facilitating the development of microbial strains capable of utilizing a wide range of substrates, such as renewable ones, thus contributing to the sustainability of the process, as will be discussed in more detail below.

### 3.2. Modification of Metabolic Pathways

#### 3.2.1. Strategies That Directly Impact the Biosynthetic Pathway of PHAs

One of the main strategies to increase PHA production is to enhance the expression of the genes involved in PHA biosynthesis, which could improve the substrate conversion efficiency. Similarly, the activity of PHA synthase will largely determine the production and type of PHAs. In addition, since the PHA depolymerase encoded by the *phaZ* gene is involved in the degradation of these bioplastics, other strategies aim to eliminate this enzyme to strengthen polymer accumulation in those strains that typically consume the bioplastic (Table 1). This combined approach was carried out in the *Rhodobacter sphaeroides* strain, in which the genes for PHB biosynthesis were overexpressed (simultaneous expression of *phaA3*, *phaB2* and *phaC1*) and the PHA depolymerase (*phaZ*) was eliminated [50]. These authors managed to increase the volumetric production of PHB by 1.7 and 3.9 times compared to the parental strain (1.88 ± 0.88 g/L PHB in nitrogen-free medium and 0.99 ± 0.05 g/L PHB in medium supplemented with 100 mM ammonium sulfate) [50]. Similarly, *P. putida* KTMQ01, which lacks *phaZ*, increased *mcl*-PHA accumulation from 66 wt% to 86 wt% compared to the starting mutant [51]. In other cases, little improvement in PHA accumulation was observed when the *phaZ* gene was eliminated, as described in *Acidovorax* sp. A1169, a psychrophilic microorganism, where no increase in PHA accumulation was reported compared to the wild type (1.22 ± 0.33 g/L PHB in the mutant strain vs. 1.71 ± 0.21 g/L PHB in the parental strain) [28]. However, this work shows the potential of these types of species in NGIB as they have low energy requirements and can be more competitive against the growth of other microorganisms.

Nevertheless, there are numerous studies in which the deletion of the *phaZ* gene has been successful. The knockout microorganism *Pseudomonas* sp. SG4502, which lacks the *phaZ* gene and in which a *tac* enhancer was introduced upstream of the PHA synthase gene (*phaC1*), showed an increased yield of 23.1% in the organism lacking only the *phaZ* gene and up to 53.8% in the mutant that also had an enhanced PHA synthase gene [43]. Greater accumulation of the bioplastic may be favored by eliminating the PHA depolymerization pathway and weakening or preventing some of the β-oxidation of fatty acids, especially those steps where intermediates necessary for PHA production are not made available. Thus, the *phzE*, *fadA* and *fadB* genes were deleted to block the native phenazine pathway and reduce β-oxidation. Additionally, *phaZ* was deleted to prevent PHA degradation, and three genes involved in PHA biosynthesis were co-overexpressed to increase carbon flux to this product [52]. The authors reported a transformed strain capable of producing 18.2 g/L biomass and accumulating 84.9 wt% *mcl*-PHA in shake flasks [52]. In another context, a similar experiment was performed by removing *phaZ* and the genes *fadBA1* and *fadBA2* in *P. putida* KT2440, and overexpressing *phaG*, *alkk*, *phaC1* and *phaC2* to boost the carbon flux toward the formation of *mcl*-PHA. This resulted in a 3.3-fold increase in *mcl*-PHA titer (116 ± 35 mg/L vs. 35 ± 5 mg/L) and a 100% increase in PHA accumulation (17.7 ± 0.2% vs. 8.9 ± 0.8%), compared to the wild-type strain when using lignin as the raw material [53].

All these data show how the elimination of PHA depolymerase favors the idea that carbon flux and energy are not directed to the depolymerization of the bioplastic, resulting in a greater accumulation of PHAs. Transformation with a vector containing the gene-encoding PHA synthase has been used several times. For instance, *Ralstonia eutropha* PTCC 1615 (currently known as *C. necator*) was transformed with a vector containing the *phaC* gene, which increased the PHB content by 1.4 times compared to the wild-type strain, yielding 7.74 ± 0.64% PHB (vs. 5.51 ± 0.43% in the parental strain), 1.32 ± 0.04 g/L biomass (1.26 ± 0.01 g/L in the wild-type) and 0.102 ± 0.080 g/L PHB (vs. 0.069 ± 0.005 g/L in the wild-type) when LB was used as medium [54]. Alternatively, the overexpression of (R)-specific enoyl-CoA hydratase (*phaJ*) together with *phaC* improves the 3-hydroxyhexanoate (3HHx) composition in the PHBHHx (poly(3-hydroxybutyrate-*co*-3-hydroxyhexanoate)) polymer, reaching 36.2 mol% [55] or even reaching values as high as 196 g/L PHBHHx with a high 3HHx (32.5 mol%) when palm kernel oil and fed-batch were employed [55]. In a similar approach, Wang and collaborators obtained a recombinant strain of *Halomonas bluephagenesis* containing a heterologous PHA synthase enzyme (PhaC_ac_) and an enoyl-CoA hydratase (PhaJ_ac_) capable of producing PHBHHx from hexanoate with and without glucose supplementation [56]. These authors reported results with great potential for industrial scaling, ranging from 0 to 37 mol% PHBHHx by modulating the glucose/hexanoate ratios, reaching 33.1 g/L biomass and 50.32 wt% P(3HB-*co*-37.23 mol% 3HHx) when fed with sodium hexanoate as the sole carbon source, or up to 51 g/L biomass with 62 wt% P(3HB-*co*-13.21 mol% 3HHx) when using a mixed carbon source [56].

#### 3.2.2. Approaches to Modulate the Amount of NAD(P)H

The synthesis of PHAs in most species is dependent on the NADPH cofactor, as it is necessary for the conversion of acetoacetyl-CoA to (R)-3-hydroxybutyryl-CoA catalyzed by the PhaB enzyme and for the conversion of 3-ketoacyl-ACP to (R)-3-hydroxyacyl-ACP catalyzed by FabG, during the synthesis of *scl*- and *mcl*-PHAs, respectively [57]. The capacity of the PHA-producing microorganism is significantly influenced by the availability of redox cofactors, which are normally derived from active metabolic pathways that provide the necessary amounts of reducing equivalents in the form of NADH and NADPH for growth and the synthesis of bioplastics. Inorganic nutrients such as phosphorus, nitrogen and oxygen cause higher NADH/NAD^+^ ratios, which to varying degrees suppress TCA cycle enzymes and favor PHA synthesis [57]. Thus, Chen and coworkers [58] used CRISPR/Cas9 technology to delete two genes involved in the TCA cycle, *sdhE* (succinate dehydrogenase assembly factor 2) and *icl* (isocitrate lyase), with the aim of increasing the amount of 3HV in the PHBV copolymer. However, given the reduced growth of the mutant strain, they constructed the mutant in *H. bluephagenesis* TY194 using a medium-strength promoter. The double mutant (Δ*sdhE*Δ*icl*) produced 17 mol% 3HV in PHBV. Furthermore, these authors constructed the mutant *H. bluephagenesis* TY194 (Δ*sdhE*, G7::Pporin-*ppc*), into which they also introduced the *ppc* gene encoding phosphoenolpyruvate carboxylase, and obtained a biomass of 6.3 g/L, an accumulation of the PHBV copolymer of 65% and 25 mol% 3HV in PHBV when cultivated in shake flasks using glucose and gluconate as carbon sources [58]. Other authors have also adopted the strategy of inhibiting the TCA cycle to increase the NADH pool and thus improve PHA content. For example, in *P. putida* KT2440, Borrero-de Acuña and colleagues [59] improved the PHA content by about 40% compared to the parental strain by overproducing the pyruvate dehydrogenase subunit AcoA in combination with the deletion of the glucose dehydrogenase gene (*gcd*). Batch bioreactor assays increased *mcl*-PHA accumulation by 120% when the mutant strain was grown on glucose, reaching 4.71 g/L biomass and 42.1 wt% *mcl*-PHA. Another alternative that favors the NADH reservoir involves the elimination of the lactate dehydrogenase (*ldh*) enzyme. By modifying *C. necator* H16 to create a knockout strain for this gene, PHB production under oxygen-limiting conditions was improved and the amount of by-products generated was reduced, increasing biomass by 31% (0.55 ± 0.44 g/L vs. 0.43 ± 0.04 g/L), PHB content by 30.9% (50.4 ± 1.1% vs. 38.6 ± 3.1%) and PHB production by 71.5% (0.28 g/L vs. 0.17 g/L in the parental strain) [60].

Increasing the NADH reservoir to favor PHB synthesis is an alternative that has been carried out by blocking the *etf* operon (electron transfer flavoprotein subunits α and β) in *H. bluephagenesis*, achieving an increase in PHB accumulation from 84% to 90% and reaching 94% accumulation when acetic acid was used along with glucose in shake flasks [61]. Even the introduction of genes from the NAD salvage pathway, such as *pncB*, favored the increase in the NAD(P)H pool. Thus, the modified strain *C. necator* NCIMB 11599 showed the highest tolerance to certain inhibitors such as furfural, which is a toxic by-product generated in some pretreatments prior to the bioconversion process. In the presence of furfural, this study showed an increase in PHB production and cell growth of 1.45 times and 1.43 times, respectively, compared to the wild-type strain [62]. However, in the absence of furfural, greater results were obtained in terms of biomass increase and PHB production, which reached 2.86 ± 0.26 g/L. In this particular case, the authors pointed out that the expression of *pncB* not only favored tolerance to certain toxic compounds such as furfural, but also increased substrate conversion, cell growth and, consequently, PHB production [62]. In view of these results, it seems interesting to control the enzymes involved in the TCA cycle, as this could somehow favor the flow of acetyl-CoA toward PHA biosynthetic pathways, where in addition the presence of NAD(P)H cofactors is essential for the proper functioning of the enzymes involved.

#### 3.2.3. Promoter Engineering to Enhance PHA Synthesis

Promoter engineering typically focuses on finding promoters that maximize the expression levels of certain genes. Promoters with different strengths are necessary for precise control of gene expression. For this reason, promoter libraries with different sequences have been developed to explore genomes based on RNAseq data and make rational designs that allow promoters to adapt to the genetic background of the microorganism that will host them. Zhang and coworkers [63] screened 30 different promoters that could improve the expression of PHA synthase genes. They selected a strong promoter and inserted it upstream of the *phaC1* gene; they also eliminated *gcd* and inserted a promoter upstream of the pyruvate dehydrogenase gene in *P. putida* KT2440. This allowed them to increase biomass, PHA accumulation and PHA production by 40% (4.06 g/L), 90% (41.93 wt%) and 165% (1.7 g/L PHA), respectively, compared to the parental strain in shake flasks [63]. Following a similar promoter engineering strategy, Zhao and colleagues [64] identified ten endogenous promoters and selected three of them to study PHA synthesis in *P. mendocina* NK-01. They engineered a recombinant strain NKU-Δ*phaZ*-16C1 in which the selected promoter was introduced upstream of the *phaC* operon and the *phaZ* gene was eliminated. This resulted in an increase from 17 to 23 wt% compared to the strain lacking only the PHA depolymerase [64]. The use of strong promoters upstream of the *phaC* operon was also used in *P. putida*, which was modified by the elimination of genomic islands and promoter engineering. Specifically, in addition to using a strong endogenous promoter upstream of *phaC*, it was also inserted upstream of the pyruvate dehydrogenase gene to increase the transcript level of PHA synthase and provide sufficient acetyl-CoA precursors [65]. The mutant strain with the most minimal KT2440 chassis (the parental strain) accumulated 55.82 wt% PHA in a 5 L fermenter, providing a good example of minimal genome cell factories [65].

Among the species of the genus *Halomonas*, the promoter of the protein with the strongest expression is the promoter of an outer membrane porin protein. The P_porin_ promoter has been used in different approaches to improve PHA production in this genus of extremophilic microorganisms. Shen and colleagues [66] constructed a promoter library based on the core region of the P_porin_. The modified *H. bluephagenesis* strain was constructed with the *orfz* gene, which encodes a 4HB-CoA transferase, under the influence of the selected promoters from the library. The best of them produced 100 g/L biomass with 80% P(3HB-*co*-11 mol% 4HB), with a productivity of 1.59 g/L·h, at laboratory scale in fed-batch mode [66]. In the same genus of bacteria, Li et al. [67] constructed a library of promoters, both constitutive and inducible, and tested the two systems in *Halomonas* TD01 to regulate the PHA biosynthetic pathway and demonstrate the utility of this expression system. Previously, in the same species, a chromosomal expression system was developed in which the expression vector of the *prpC* gene (2-methylcitrate synthase) also contained the P_porin_, and in which they achieved a modified *Halomonas* TD01 strain lacking the *prcpC* gene. In particular, in this study, they used the mutant strain to which they added the triple knockout for three *phaZ* genes, in which they carried out a pilot-scale test in a 500 L fermenter, achieving a biomass accumulation of 112 g/L with 70 wt% PHB when using glucose as a carbon source, and a biomass of 80 g/L with 70 wt% P(3HB-*co*-8 mol% 3HV) when supplemented with propionic acid [68].

**Table 1:** Enhancing PHA production by modifying metabolic pathways using engineered microorganisms. The table shows the type of PHA produced, the PHA content (wt%) and concentration (g·L^−1^), the biomass produced (as dried cell weight—DCW—g·L^−1^) and the production scale. nd.: not determined in the study; approx.: approximately.

Bacterial Strain	PHA Type	PHA Cont. (wt%)	PHA Conc. (g·L^−1^)	Yield (g·g^−1^)	DCW (g·L^−1^)	Production Scale	Ref.
*Rhodobacter sphaeroides* HJ Δ*phaZ(phaA3/phaB2/phaC1)*	PHB	79.8 ± 6.0	1.88 ± 0.08	nd.	2.37 ± 0.08	Shake flasks	[50]
*Pseudomonas putida KT2442* Δ*phaZ*	*mcl*-PHA	86.47 ± 4.90	nd.	nd.	4.02 ± 0.01	Shake flasks	[51]
*Acidovorax* sp. A1169Δi*-phaZ*	PHB	nd.	1.22 ± 0.03	nd.	nd.	Shake flasks	[28]
*Pseudomonas* sp. SG4502 +(*tac-phaC2*)	*mcl*-PHA	nd.	nd.	0.04	1.83	Shake flasks	[43]
*Pseudomonas* sp. SG4502 Δ*phaZ*	*mcl*-PHA	nd.	nd.	0.032	1.44	Shake flasks	[43]
*Pseudomonas chlororaphis* HT66 *HT4*Δ*::C1C2J*	*mcl*-PHA	84.9	15.5	nd.	18.2	Shake flasks	[52]
*Pseudomonas putida* KT2440 Δ*phaZ/fadBA1/fadBA2*	*mcl*-PHA	17.7	0.116	nd.	0.654	Shake flasks	[53]
*Ralstonia eutropha* PTCC 1615 *phaC*	PHB	7.74 ± 0.64	0.102 ± 0.080	nd.	1.32 ± 0.04	Shake flasks	[54]
*Cupriavidus necator* H16 CnTRCB/dbktB/dA1528/pCTRP-NSDG	PHBHHx	57.1 ± 2.7 (with 36.2 ± 0.4 mol% 3HHx)	6.4 ± 0.1	nd.	11.2 ± 0.6	Shake flasks	[55]
*Halomonas bluephagenesis* G34Δ*phaC_td_*/*fadB_1_*	P(3HB-*co*-13.21 mol% 3HHx)	62	nd.	nd.	51	7 L-reactor	[56]
*Halomonas bluephagenesis* Δ*sdhE*Δ*icl*	PHBV	approx. 90	nd.	0.44	approx. 10	Shake flasks	[58]
*Halomonas bluephagenesis* Δ*sdhE*, G7::Pporin-*ppc*	PHBV (25 mol% 3HV)	65	nd.	nd.	6.3	Shake flasks	[58]
*Pseudomonas putida* KT2440 Δ*gcd*-*acoA*	*mcl*-PHA	42.1	nd.	nd.	4.71	2 L reactor	[59]
*Cupriavidus necator* H16 Δ*ldh*-*vgb*	PHB	50.4 ± 1.1	0.28	nd.	0.55 ± 0.44	Shake flasks	[60]
*Halomonas bluephagenesis* Δ*etf-x-β*	PHB	94	10.46 ± 0.27	nd.	11.47 ± 0.03	Shake flasks	[61]
*Cupriavidus necator* NCIMB 11599 *+pncB*	PHB	nd.	2.86 ± 0.26	nd.	approx. 3.5	Shake flasks	[62]
*Pseudomonas putida* KT2440 KTU-P46C1A-Δ*gcd*	*mcl*-PHA	41.93	1.7	nd.	4.06	Shake flasks	[63]
*Pseudomonas mendocina* NK-01 NKU-Δ*phaZ*-16C1	*mcl*-PHA	23	nd.	nd.	approx. 1.5	Shake flasks	[64]
*Pseudomonas putida* KT2440 KTU-U27Δ*gcd-P46CA*	PHA	55.82	3.01	nd	5.4	5 L reactor	[65]
*Halomonas bluephagenesis* + *orfz*	P(3HB-*co*-11 mol% 4HB)	79.5	nd.	nd.	100.3	Shake flasks	[66]
*Halomonas* spp. Δ*phaZ*Δ*prpC-*	PHB	70	nd.	nd.	112	500 L reactor	[68]
*Halomonas* spp. Δ*phaZ*Δ*prpC-*	P(3HB-*co*-8 mol% 3HV)	70	nd.	nd.	80	500 L reactor	[68]

### 3.3. Expanding the Range of Renewable Substrates Bioconverted into PHAs

The use of renewable substrates, such as lignocellulosic residues, starch and other carbohydrates, protein hydrolysates, oils and fats, among others, not only reduces production costs but also contributes to the sustainability of the process by reusing waste and minimizing the dependence on fossil resources. However, in many cases, the use of these types of raw materials requires a pretreatment step in the process to make the molecules that will be used as the carbon source in the bioconversion process accessible to PHAs. This type of pretreatment usually requires a high energy demand, resulting in a lower achievement in reducing production costs [32]. For this reason, the use of microbial biotechnology to achieve the direct use of feedstocks in PHA production can be an alternative that makes it possible to reach the goal of cost reduction (Table 2).

Regarding the use of starchy substrates for PHA production, Bhatia and colleagues [69] achieved the co-expression of the genes for a functional amylase from *Panibacillus* sp. and the PHA biosynthesis genes from *R. europha* (currently *C. necator*) in *Escherichia coli*. In this work, starch could be used as the sole carbon source to produce 1.24 g/L of PHB with an accumulation of 57.4% PHB in shake flasks [69]. The *C. necator* strain itself was also modified by introducing the genes for glucodextranase *G1d* from *Arthrobacter globiformis* I42 and α-amyase *amyZ* from *Zunongwangia profunda* SM-A87. The recombinant strain with amylolytic activity produced 13.29 ± 0.98 g/L biomass, 5.78 ± 0.82 g/L PHB and 43.32 ± 3.18% PHB from broken rice [70]. Recently, the co-expression of the α-amylase gene *amy03713* from *Vibrio alginolyticus* LHF01 with a strong broad-spectrum promoter (P_pdc_) and the glucosidase *glu04552* in *H. bluephagenesis* was reported [71]. In addition, the expression of the *amy03713* gene was optimized by replacing the natural RBS (ribosome binding site) sequence with a stronger one. In this situation, a biomass production of 11.26 g/L was achieved and 56.22% PHB was accumulated intracellularly at a concentration of 6.32 g/L PHB in shake flasks [71].

Molasses is another common substrate with great potential for PHA production. However, to be directly metabolized, it is necessary to assimilate sucrose, an ability that the native *C. necator* strain lacks. To overcome this drawback, microbial biotechnology was used to introduce sucrose utilization genes (*csc*) into the bacterial genome. These *E. coli* genes were introduced into recombinant strains of *C. necator*. Depending on the set of genes selected for transformation, the recombinant strains produced PHB or PHBHHx [72]. In particular, the one containing the crotonyl-CoA reductase (*ccr*), ethylmalonyl-CoA decarboxylase (*emd*) and other modifications produced PHBHHx with a maximum amount of 3HHx of 27 mol%. Furthermore, when this strain was cultivated in fed-batch mode, the amount produced increased, yielding PHBHHx with an amount of 3HHx of approximately 4 mol% and 113 g/L [72]. In another study, recombinant strains of *R. eutropha* (*C. necator*) expressing the *sac* gene (fructofuranosidase) from *Mannheimia succiniciproducens* were constructed. In this study, the authors were unable to grow the modified strains on crude sugarcane molasses; however, after treatment with activated charcoal to remove inhibitory compounds, the strain produced 82.5 wt% PHB in batch [73].

The assimilation of lipid substrates for conversion to PHAs involves the metabolic pathways of fatty acid β-oxidation or *de novo* synthesis [74,75]. Microbial biotechnology can be used to redirect the carbon flux toward these pathways, which will generate precursors mainly for *mcl*-PHAs. In this regard, the review by Talan and colleagues [75], as well as by Wang and coworkers [74], summarize numerous examples in which recombinant strains capable of utilizing oily residues of both plant and animal origin have been developed. In many of these approaches, lipase-related genes were introduced and the resulting recombinant strains showed high extracellular enzymatic activity, while in other studies the strategy was to modify β-oxidation genes. Recently, the case of the species par excellence in PHA production, *C. necator* modified with the *phaCBP-M-CPF4* gene, has been reported. The best producing strain yielded 8.5 g/L biomass, 75.5 wt% PHA and 6.4 g/L PHA when waste cooking oil was used as a carbon source [76]. Other strategies have been pursued as approaches to continuous feeding that could be compatible with more industrial production. In particular, it is shown how the recombinant strain *R. eutropha* Re2058/pCB113, a modified strain expressing the heterologous gene of the PhaC enzyme, accumulated 80% PHA at a titer of 45 g/L [77].

On the other hand, lignocellulose-based residues, such as wheat straw, sugarcane bagasse or forest residues, among others, represent the most abundant and renewable carbon source for PHA production. Their massive availability and low cost make them attractive substrates for bioconversion into biopolymers. Lignocellulose, composed of cellulose, hemicellulose and lignin, provides a supply of fermentable sugars after appropriate pretreatment and enzymatic digestion. However, its bioconversion to PHAs is limited by the complex structure of lignin, which hinders the release of fermentable sugars, and degradation products that can inhibit microbial growth [78]. Genetic engineering has helped improve the bioconversion process from these types of residues, as can be seen in some detailed reviews on the subject, where some of the most recent examples in this field are indicated [74,79]. For example, Oliveira-Filho and collaborators [80] managed to increase the accumulation of the copolymer P(3HB-*co*-3HHx) up to 55% with 3HHx mol%, ranging from 8 to 35 mol% from a carbon source of xylose and hexanoate. Another recent example in *Halomonas* sp. Y3 shows how the modification of this strain through the high secretion apparatus of laccase made it possible to obtain up to 1314 mg/L of PHA from lignin streams under non-sterile conditions [11]. Although microbial biotechnology has made significant progress in this area, much remains to be done to improve and optimize the bioconversion process and to achieve the expression of lignocellulolytic enzymes and efficient PHA production in the same microorganism.

Apart from the aforementioned renewable substrates, engineered microorganisms can also directly utilize additional feedstocks like glycerol and C1 carbon sources (such as CO_2_, CO or even CH_4_) as carbon sources to produce PHAs. The use of these substrates is not only an effective strategy to valorize industrial waste and reduce production costs, but also contributes to the mitigation of greenhouse gases by using CO_2_ or other gases as raw materials [74].

**Table 2:** Enhancing PHA production using engineered microorganisms from different carbon substrates. The table shows the carbon source used, the type of PHA produced, the PHA content (wt%) and concentration (g·L^−1^), the biomass produced (as dried cell weight—DCW—g·L^−1^) and the production scale. nd.: not determined in the study; approx.: approximately.

Bacterial Strain	Carbon Source	PHA Type	PHA Cont. (wt%)	PHA Conc. (g·L^−1^)	Yield (g·g^−1^)	DCW (g·L^−1^)	Production Scale	Ref.
*Escherichia coli + pha genes + amylase*	Starch	PHB	57.4	1.24	nd.	approx. 2.1	Shake flasks	[69]
*Cupriavidus necator* DSM 545 + *G1d* + *amyZ*	Broken rice	PHB	43.32 ± 3.18	5.78 ± 0.82	nd.	13.29 ± 0.98	Shake flasks	[70]
*Halomonas bluephagenesis* TD01/p341-*amy03713-glu04552*	Starch	PHB	56.22	6.32	nd.	11.26	Shake flasks	[71]
*Halomonas bluephagenesis* TD01/p341-*amy03713-glu04552*	Starch	P(3HB-*co*-3HV)	53.75	4.91	nd.	9.14	Shake flasks	[71]
*Halomonas bluephagenesis* TD01/p341-*amy03713-glu04552*	Starch	P(3HB-*co*-4HB)	50.89	5.33	nd.	10.48	Shake flasks	[71]
*Cupriavidus necator* + *cscA* + *cscB* + *emd* + *ccr*	Sucrose	P(3HB-*co*-4 mol% 3HHx)	nd.	113	0.40	approx. 120	5 L reactor	[72]
*Ralstonia eutropha* + *sacC*	Sugarcane molasses	PHB	82.5	16.8	nd.	20.3	2.5 L reactor	[73]
*Cupriavidus necator Re2058/pHT1-CBP−M−CPF4*	Waste cooking oil	PHA	75.5	6.4	nd.	8.5	Shake flasks	[76]
*Ralstonia eutropha Re2058/pCB113*	Waste pork fat	PHA	80	45.6	nd.	57	Shake flasks	[77]
*Ralstonia eutropha Re2058/pCB113*	Waste pork fat	PHA	70	31.5	nd.	45	150 L reactor	[77]
*Burkholderia sacchari* + *xylA* + *xylB* + *tktA* + *phaC*	Xylose + hexanoate	P(3HB-*co*-20.91 mol% 3HHx)	55.30	nd.	0.51	21.91	5 L reactor	[80]
*Halomonas* sp. Y3 + laccase secretion	Lignin	PHA	58.20 ± 3.64	0.693 ± 0.015	nd.	0.119 ± 0.059	Shake flasks	[11]

### 3.4. Improvement in the Downstream Processing

Microbial biotechnology has significantly improved the downstream processing of PHAs through genetic modification of the producing microorganisms, primarily based on enhancing cell morphology and PHA secretion. Genetic modifications to improve cell morphology allow for a greater accumulation of PHA granules [74], which in many cases enables a more efficient separation of PHA-producing cells from the culture medium and eventually makes the polymer easier to recover and purify at lower cost [81]. The small size of the bacteria and the stiffness of their cell wall, however, are significant constraints on cell morphology [82]. These factors limit the amount of polymer the bacteria can accumulate and raise the cost of extraction because it is difficult to break down the cell wall. To overcome these limitations, some microbial biotechnology strategies focus on using genes related to cell morphology or involved in cell wall synthesis. By modifying these genes, it is possible to alter the structure and composition of the cell wall, making it more flexible or allowing for a larger cell size, which in turn can increase PHA accumulation and facilitate its extraction.

SulA is an inducible protein that inhibits cell division by interacting with the FtsZ protein [83]. Overexpression of the *sulA* gene in rod-shaped *E. coli* BL21 caused these cells to become filamentous, increase intracellular space and produce more PHB, increasing bioplastic content and the biomass produced by more than 100% compared to the control [83]. Furthermore, the overexpression of *sulA* in modified *E. coli* strains capable of producing P(3HB-*co*-4HB) resulted in higher polymer production. Additionally, this work demonstrated that these modified filamentous bacteria aided in subsequent processing by allowing for cell separation from the broth by gravity precipitation. Gravity separation has proven to be a cost-effective alternative in downstream separation processes [81]. The overexpression of *sulA* in the modified *E. coli* strain *JM109*Δ*minCD* (in which the cell fission ring—Z-ring—location genes *minCD* were eliminated), which is capable of synthesizing PHB because it also contains the *phbCAB* synthesis operon, leads to these cells becoming long filaments and to an increase in PHB accumulation over wild-type strains. In shake flasks, this strain grown in multiple fission pattern accumulated 70% PHB and 9 g/L biomass, higher than the corresponding control which accumulated 51% PHB and 8 g/L cell dried weight [84]. Zhao et al. [85] investigated the elimination or overexpression of several genes related to cell morphology and division in a more recent study, including the *minCD* genes, the peptidoglycan degradation gene *nlpD*, the actin-like cytoskeleton protein gene *mreB*, the Z-ring formation gene *ftsZ* and the FtsZ inhibitor gene *sulA*. The best results were obtained when *ftsZ* was overexpressed in *P. mendocina* NKU, and these authors were able to increase biomass by 23.26% and *mcl*-PHA accumulation by 60.87% (from 18.09 wt%—0.23 g/L to 23.69 wt%—0.37 g/L) [85].

In other cases, it has been demonstrated how smaller cells can accumulate a greater number of PHB granules by decreasing their size through increased surface area, thanks to genes encoding phasins, the surface-binding proteins of PHA granules. Thus, Lee et al. [86] studied the effect of heterologous phasins and determined the best synergistic combination of *phaP* genes to produce PHB. They introduced a combination of genes, *phaP1* and *phaP3*, from *Halomonas* sp. YLGW01 into *E. coli*. The resulting strain increased PHB production 2.9-fold, resulting in an up to 65% increase in PHB and up to 48% increase in biomass accumulation [86]. Weakening cell rigidity also favors the accumulation of more PHAs, as seen in the work of Zhang and colleagues [87], who used the CRISPRi system to downregulate 10 genes involved in the cell wall synthesis pathway to obtain a modified *E. coli* strain that was more elastic and had more space for PHB accumulation, reaching 93% PHB, a much higher value than the modified strain with only a thinner cell wall [87]. The elimination of genes encoding exopolysaccharide (EPS) is beneficial for PHA synthesis, as it reduces downstream costs due to rapid self-flocculation and cell lysis [88]. In this study, 85% PHA and 96 g/L biomass were produced when production was scaled up to a 5000 L bioreactor. Furthermore, these authors proposed a specific reactor configuration in which they reduced the stirring speed by 50% and air intake by 25%, as well as the time required for centrifugation to separate cells from the broth [88].

Moreover, genetic engineering can enable the secretion of PHAs directly into the culture medium, which is a significant innovation and eliminates the need for cell rupture and extraction processes, greatly simplifying downstream processing. This not only reduces operating costs, but also increases the overall yield of PHA production. Recently, Zhang and colleagues [89] provided a valuable review of different technologies for PHA recovery. They highlighted systems such as yeast surface display (YSD), which allows target compounds to be synthesized directly outside of the cell, or other cutting-edge lipid-based excretion systems [89] that facilitate PHA recovery and contribute to a more sustainable and economically viable production process.

## 4. Conclusions

In summary, microbial biotechnology has emerged as a critical tool to overcome the limitations in the industrial production of PHAs. The modification of biosynthetic pathways to optimize carbon flux, the use of a wide range of renewable substrates and the improvement in downstream processing are significant advances that can reduce costs and increase the competitiveness of PHAs compared to conventional plastics. However, it is still far from being developed on an industrial scale due to ongoing challenges, such as ensuring the long-term durability of genetic modifications. In the future, it is essential to continue to explore new genetic engineering techniques and process optimizations to face these challenges. The integration of emerging technologies, such as CRISPR/Cas, may open new opportunities for the efficient and sustainable production of PHAs. In addition, collaboration between academic research and industry will be key to the scalability and commercialization of these biopolymers, thus contributing to a circular economy and reducing the dependence on fossil resources.
